# A pharmacogenetic study of perampanel: association between rare variants of glutamate receptor genes and outcomes

**DOI:** 10.3389/fgene.2023.1215493

**Published:** 2023-11-24

**Authors:** Chih-Hsiang Lin, Chen-Jui Ho, Shih-Ying Chen, Yan-Ting Lu, Meng-Han Tsai

**Affiliations:** ^1^ Department of Neurology, Kaohsiung Chang Gung Memorial Hospital and Chang Gung University College of Medicine, Kaohsiung, Taiwan; ^2^ Genomics and Proteomics Core Laboratory, Kaohsiung Chang Gung Memorial Hospital and Chang Gung University College of Medicine, Kaohsiung, Taiwan; ^3^ Medical School, College of Medicine, Chang Gung University, Taoyuan, Taiwan

**Keywords:** perampanel, glutamate receptor gene, rare genetic variants, drug response, adverse drug reaction, 1-year retention

## Abstract

**Introduction:** The selection of antiseizure medication usually requires a trial-and-error process. Our goal is to investigate whether genetic markers can predict the outcome of perampanel (PER) use in patients with epilepsy.

**Method:** The studied participants were selected from our previous epilepsy genetics studies where whole exome sequencing was available. We reviewed the medical records of epilepsy patients older than 20 years old treated with PER. The outcome of PER treatment included the response to PER, the occurrence of any adverse drug reaction (ADR), the presence of behavior ADR, and the ability to adhere to PER for more than 1 year. We investigated the association between the rare variants of the glutamate receptor genes and the outcomes of PER use.

**Result:** A total of 83 patients were collected. The gene group burden analysis showed that enriched genetic variants of the glutamate receptor gene group were statistically significantly associated with the occurrence of ADR, while the glutamate ionotropic receptor delta type subunit had a nominal association with the occurrence of ADR. The gene collapse analysis found that *GRID1* had a nominal association with the occurrence of ADR and *GRIN3A* had a nominal association with the occurrence of behavior ADR. However, these nominal associations did not remain statistically significant once adjusted for multiple testing.

**Discussion:** We found that enriched rare genetic variants of the glutamate receptor genes were associated with the occurrence of ADR in patients taking PER. In the future, combining the results of various pharmacogenetic studies may lead to the development of prediction tools for the outcome of antiseizure medications.

## Introduction

The treatment of epilepsy requires the use of antiseizure medications (ASMs) to cease or control seizures. To choose the optimal ASMs, the treating physicians often consider the patient’s seizure types, age, gender, co-medications, and comorbidities (Perucca and Tomson, 2011) before offering the ideal drug. However, the response to ASMs is unpredictable and the treating physician can only balance the effect and adverse drug reactions (ADRs) of ASMs in a trial-and-error process, which relies heavily on the physician’s judgment and experience. During this process, the patient would suffer from inadequate seizure control from low ASM dose to prevent ADR or endure unpleasant ADR due to the rapid titrating of ASM to control the seizures. There is an unmet need for biomarkers that can guide or suggest the optimal ASM for individual patients to achieve the goal of “no seizures, no side effects” (Glauser, 2011).

Perampanel (PER) is a non-competitive α-amino-3-hydroxy-5-methyl- 4-isoxazole propionic acid (AMPA) receptor antagonist (Plosker, 2012) that inhibits the excitatory neurotransmission to achieve its antiseizure activity (Rogawski and Hanada, 2013). PER is a new-generation ASM that effectively controls both focal and generalized seizures (Tsai et al., 2018). It had the advantage of few drug-drug interactions (Kwan et al., 2015) and long potency (Villanueva et al., 2016a), making it easier to use for physicians and increasing adherence for patients. One of its drawbacks is behavior disturbance including irritation, aggression, or other psychotic symptoms (Fong et al., 2022). Hence, an objective indicator predicting the outcome of PER use may greatly enhance the ability of treating physicians to achieve seizure control and minimize ADRs.

The advance of massively parallel genetic sequencing has expanded our understanding of the relationship between genetic factors and phenotype. This technology allows the incorporation of pharmacogenomic data into the clinical decision-making process (Balestrini and Sisodiya, 2018). One example was the association of HLA-B*1502 and the development of Stevens-Johnson syndrome/toxic epidermal necrolysis in Taiwanese and Southeast Asian populations (Chung et al., 2004) that led physicians to perform a genetic test before prescribing aromatic ASMs to these ethnic groups to prevent severe skin ADR. The association between ASM responsiveness and genetic variants was not as robust as that for dermatological side effects. The association between the responsiveness of sodium channel-blocking ASMs and sodium channel genes is by far the most extensively studied but the results are inconsistent among different ethnic groups (Abe et al., 2008; Sanchez et al., 2010; Manna et al., 2011; Haerian et al., 2013; Kumari et al., 2013; Ma et al., 2014; Angelopoulou et al., 2017; Lin et al., 2021). A large study conducted by EpiPGX Consortium suggested the resistance of ASM may be related to damaging genetic variants (Wolking et al., 2020a). The accumulation of data from these pharmacogenetic studies may 1 day provide robust genetic data to assist the decision-making of physicians in prescribing ASM.

We hypothesize that genetic variants in the glutamate receptor genes may affect the binding between PER and the glutamate receptors or interfere with the interaction of various subtypes of glutamate receptors. These alternations change the downstream neurotransmission and result in differences in the outcome of PER treatment. Currently, no research has focused on the association between the genetic variants in glutamate receptors and the effect of PER. In this study, we searched the pharmacogenomic relationship between genetic variations of the glutamate receptors and the outcome of PER treatment.

## Material and methods

### Ascertainment of subjects

The studied participants were selected from our previous epilepsy genetics studies where whole exome sequencing (WES) was available. All participants are Taiwanese aged more than 20 years old and provided written, informed consent before sequencing. The studies were approved by the Chang Gung Medical Foundation Institutional Review Board.

Patients with a history of psychogenic nonepileptic seizures were excluded due to the difficulty in determining reliable seizure frequency. Clinical data including gender, onset age of seizure, types of seizure, etiology of seizure, seizure frequency, other medical diseases, the types and maintenance dose of ASMs, ADRs, electroencephalograms, and brain images were collected.

### Outcomes related to perampanel treatment

We defined four separate outcomes regarding the result of using PER, which were the response to PER, the occurrence of ADR, the presence of behavior ADRs, and the 1-year retention of PER. These outcomes represent different aspects of treating with PER. The response to PER was to evaluate its effectiveness in controlling seizures. The ADRs and behavior ADRs are annoying side effects to the patient, caregiver, and healthcare provider, which may result in a decreased quality of life or withdrawal of the medication (Hansen et al., 2018). The 1-year retention denotes a balance between the effects and side effects of PER, patients with acceptable seizure control and tolerable side effects may have better adherence to the medication being used (Im et al., 2021).

The response to PER was defined according to the seizure frequency after its prescription and categorized according to the International League Against Epilepsy (ILAE) consensus (Kwan et al., 2010) and EpiPGX Consortium (Androsova et al., 2017; Wolking et al., 2020b). Responding to PER was defined as seizure-free under ongoing PER treatment for at least 1 year and before initiation of any other treatment. Failure to respond to PER was defined as still having seizures during the use of PER or the need for initiation of any other treatment. The occurrence of ADR was defined as PER-related adverse events (Tsai et al., 2018) both occurring after PER use and deemed to be caused by PER by the treating physician. The PER-related ADRs included dizziness, somnolence, headache, fatigue, behavior problems, and falls (Steinhoff et al., 2013; Leppik et al., 2017). The presence of behavior ADRs was defined as irritation, violent behavior, and/or psychotic symptoms that occurred after PER use and were considered caused by PER by the treating physician. One-year retention was defined as patients who kept PER use for more than 1 year.

### Gene group and genotype

We specifically focus on the glutamate receptor genes obtained from the HUGO Gene Nomenclature Committee at the European Bioinformatics Institute (https://www.genenames.org/) (Seal et al., 2023). The glutamate receptor gene list includes 26 genes, *GRIA1-4*, *GRID1-2*, *GRIK1-5*, *GRIN1, GRIN2A-D, GRIN3A-B*, and *GRM1-8,* which is further separated into five functional subunit gene groups.

We followed the grouping method of HUGO, including 1. The glutamate ionotropic receptor AMPA type subunit (*GRIA1-4*), 2. The glutamate ionotropic receptor delta type subunit (*GRID1-2*), 3. The glutamate ionotropic receptor kainate type subunit (*GRIK1-5*), 4. The glutamate ionotropic receptor N-methyl-D-aspartate (NMDA) type subunit (*GRIN1, GRIN2A-D,* and *GRIN3A-B*), and 5. The glutamate metabotropic receptor (*GRM1-8*).

### Statistical analysis

In our study, we focused on the rare variants of the glutamate receptor genes and defined them as minor allele frequency (MAF) ≦ 0.05 in gnomAD (https://gnomad.broadinstitute.org/) (Karczewski et al., 2020). We excluded all synonymous single nucleotide variants.

First, we conduct a gene group burden analysis to search for the different enrichment of rare variants in the gene group that might be associated with the outcome of PER use. This was done by testing the association between the presence/absence of phenotypes and the number of rare variants in each gene group using the Mann–Whitney *U* test. Second, a gene collapse analysis was performed to find the association between the presence/absence of phenotypes and the presence/absence of at least one rare variant in each glutamate receptor gene using the Fisher exact test. Significance was defined as *p*-values of less than 0.05 but to cope with the problem of multiple testing, we adjusted *p*-values by the Bonferroni correction. The clinical statistics were presented as percentages for categorical data and median plus interquartile range for skewed continuous variables.

## Results

### Demographic characteristics

Overall, 83 Taiwanese patients who took PER for seizure control were collected for this study. The demographic data is presented in Table 1. In the study cohort, we included 35 females and 48 males. There were 76 patients (91.6%) with focal seizures, four (4.8%) generalized seizures, and three (3.6%) unclassified. The etiology of seizure included genetic in seven patients (8.4%), immunologic in four (4.8%), infectious in four (4.8%), metabolic in one (1.2%), structural in 33 (39.8%), and unknown in 34 (41.0%). The age of seizure onset was 13.5 (interquartile range (IQR) = 6.0–23.0) years old and the age at PER use was 37.0 (IQR = 28.0–46.0) years old. Ten patients had an underlying intellectual disability and one with an autism spectrum disorder. The interval from seizure onset to the use of PER was 22 (IQR = 8.5–33) years and the duration of PER use was 885 (IQR = 339.5–1626.5) days. The median number of ASMs used before PER was 4 (IQR = 2–6) and three patients underwent non-medical treatment before the use of PER. One had callosotomy, another received deep brain stimulation, and the other received vagus nerve stimulation. In our cohort, 14 patients were responding to PER, and 69 failed; 22 patients had ADRs and 61 did not; 14 patients had behavior ADRs and 69 did not; a total of 61 patients were able to adhere to PER for more than 1 year, and 22 were not. Among patients with ADRs, five had dizziness, three had somnolence, and 14 had behavior ADRs. Behavior ADR occurred in two patients with underlying intellectual disability and one with an autism spectrum disorder. There were no patients with psychiatric histories before PER use in the study cohort. Neither the types of seizure, the etiologies of seizure, nor the interval from seizure onset to the use of PER was associated with the outcome of perampanel use, the result was presented in Supplementary Table S1–S3, respectively.

**TABLE 1:** The demographic data of our study cohort.

	N = 83
Seizure onset age (years)	13.5 (6.0–23.0)
Male	48 (57.8%)
Female	35 (42.2%)
Seizure type	
Focal	76 (91.6%)
Generalized	4 (4.8%)
Unspecified	3 (3.6%)
Age at the use of PER (years)	37.0 (28.0–46.0)
Duration of PER use (days)	885 (339.5–1626.5)
Number of patients responding to PER	14 (16.9%)
Number of patients not responding to PER	69 (83.1%)
Number of patients with ADRs	22 (26.5%)
Number of patients without ADRs	61 (73.5%)
Number of patients with behavior ADRs	14 (16.9%)
Number of patients without behavior ADRs	69 (83.1%)
Number of patients who adhere to PER for ≥1 year	61 (73.5%)
Number of patients who adhere to PER for <1 year	22 (26.5%)

Continuous variables were presented as median (interquartile range).

Categorical variables were presented as n (%).

Abbreviations: ADR, adverse drug reaction; PER, perampanel.

### Burden analysis of glutamate receptor genes and outcomes related to PER

**FIGURE 1:**
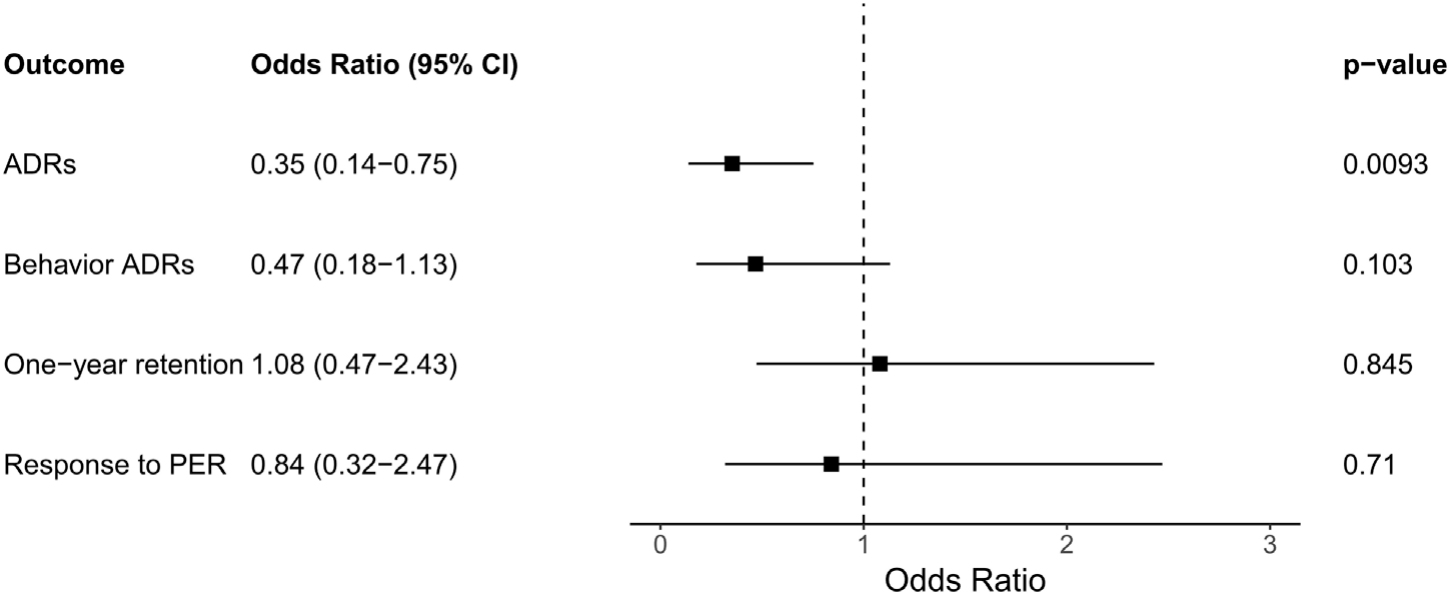
The association of the outcomes and the enrichment of rare variants in the glutamate receptor gene group. Abbreviations: ADR, adverse drug reaction; CI, confidence interval; PER, perampanel.

We first analyzed the association between the rare variants of the glutamate receptor gene group, which includes 26 genes, and the outcomes of PER therapy. The result showed that enriched genetic variants of the glutamate receptor gene group were statistically significant associated with the occurrence of ADR (OR = 0.35, CI = 0.14–0.75, *p =* 0.0093; Figure 1). No statistically significant association between the enrichment of rare variants and the response to PER, behavior ADR, or 1-year retention (Figure 1).

**FIGURE 2:**
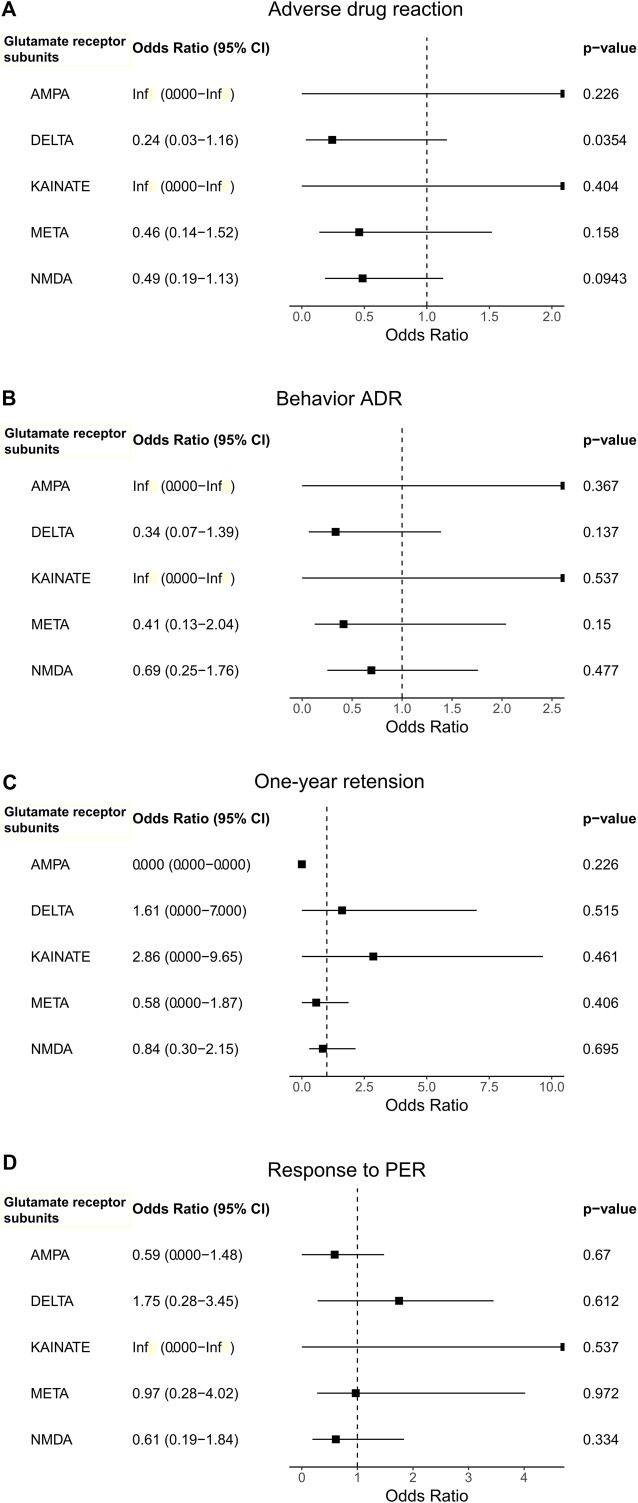
The association between outcomes [**(A)**. Adverse drug reaction, **(B)**. Behavior ADR, **(C)**. One-year retension, and **(D)**. Response to PER)] with the use of perampanel and the enrichment of rare variants in the glutamate receptor subunit gene groups.

Next, we performed burden analysis to find associations between the rare variants of each of the five function sub-groups (AMPA, NMDA, delta, kainate, and metabotropic) and the outcomes of PER treatment. The occurrence of the ADR had a nominal association with having more rare variants in the glutamate ionotropic receptor delta type subunit compared to those without ADRs (OR = 0.24, CI = 0.03–1.16, *p =* 0.0354; Figure 2A.). With five function sub-groups in the analysis, Bonferroni correction was applied to correct for multiple testing, defining a *p*-value threshold for significance of 0.01. Therefore, this nominal difference did not remain statistically significant after the Bonferroni correction. Neither the response to PER, the presence of behavior ADR, nor the 1-year retention of PER was associated with the enrichment of rare variants in any of the five subgroups (Figure 2).

### Gene collapse analysis of glutamate receptor genes and phenotypes related to PER

**FIGURE 3:**
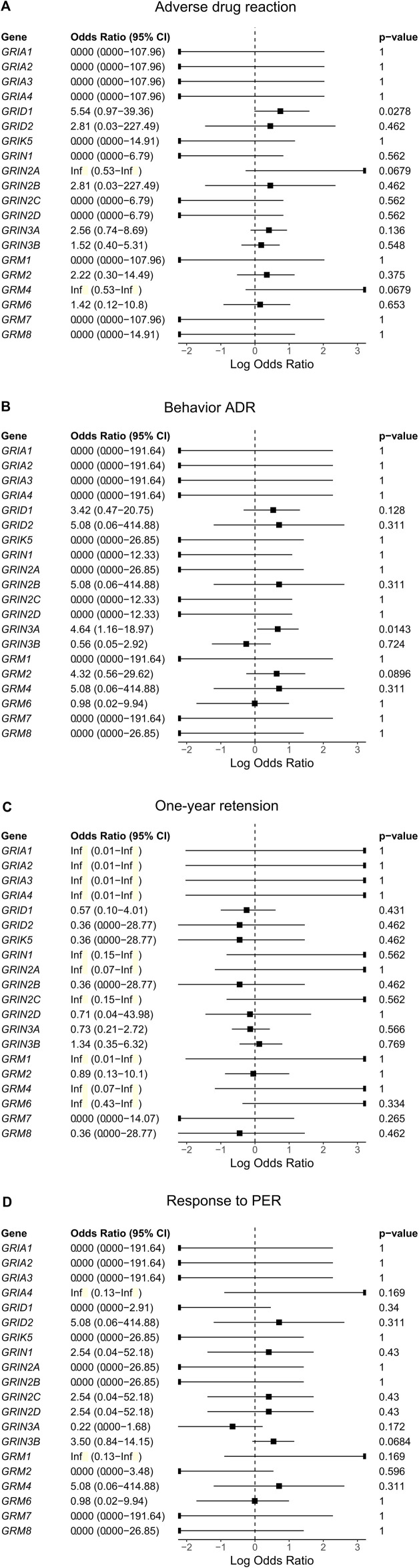
The association between rare variants of glutamate receptor genes and the outcomes [**(A)**. Adverse drug reaction, **(B)**. Behavior ADR, **(C)**. One-year retension, and **(D)**. Response to PER)] of using perampanel (PER).

We performed a gene collapse analysis to find the association between the presence of the rare variants in the glutamate receptor genes and the outcomes of PER use. In terms of ADR, the presence of rare variants in *GRID1* had a nominal association with the occurrence of ADR (OR = 5.54, CI = 0.966–39.36, *p* = 0.0278, Figure 3A) compared to those without. Two variants were observed, *GRID1*:c.1585G>A and *GRID1*:c.1287_1288delinsGG. *GRID1*:c.1585G>A results in changing the amino acid from valine to isoleucine (NM_017551.3:p(Val529Ile)) in the agonist binding domain (ABD) of GluD1. *GRID1*:c.1287_1288delinsGG results in a non-frameshift substitution of methionine to valine (NM_017551.3:p(Met430Val)) between the N-terminal domain (NTD) and ABD of GluD1.

For behavior ADRs, the presence of rare variants in *GRIN3A* had a nominal association with the occurrence of behavior ADR (OR = 4.64, CI = 1.16–18.97, *p* = 0.0143, Figure 3B) compared with those without. Five variants were identified, including *GRIN3A*:c.55C>T, *GRIN3A*:c.322C>G, *GRIN3A*:c.1438C>G, *GRIN3A*:c.1462A>G, and *GRIN3A*:c.2501_2502delinsCA. *GRIN3A*:c.55C>T results in changing the amino acid from proline to serine (NM_133445.3:p(Pro19Ser)) in the N-terminal signal peptide of GluN3A. *GRIN3A*:c.322C>G results in changing the amino acid from proline to alanine (NM_133445.3:p.(Pro108Ala)) in the NTD of GluN3A. *GRIN3A*:c.1438C>G, and *GRIN3A*:c.1462A>G result in changing the amino acid from arginine to glycine (NM_133445.3:p.(Arg480Gly)) and lysine to glutamate (NM_133445.3:p.(Lys488Glu)), respectively, in the ABD of GluN3A. *GRIN3A*:c.2501_2502delinsCA results in a non-frameshift substitution of aspartate to asparagine (NM_133445.3:p.(Asp834Asn)) in the transmembrane domain of GluN3A.

Rare variants of the glutamate receptor genes were neither associated with the response to PER nor 1-year retention (Figure 3).

With 26 genes involved in our gene collapse analysis, Bonferroni correction was again applied to correct for multiple testing, defining a *p*-value threshold for significance of 1.92*10^–3^. As a result, none of the above-mentioned nominal associations remain statistically significant after the Bonferroni correction.

**FIGURE 4:**
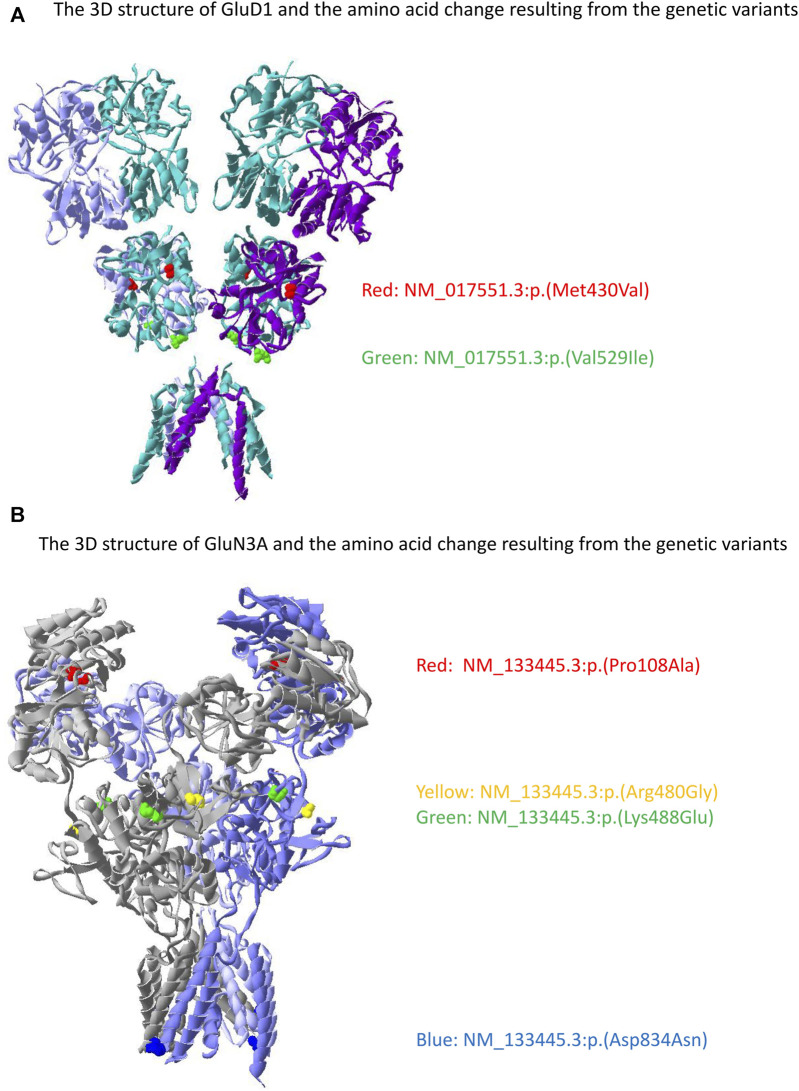
The location of the genetic variant in *GRID1* and *GRIN3A* with its corresponding amino acid change on GluD1 and GluN3A proteins. **(A)** Two variants were identified in GluD1 with the red denotes NM_017551.3:p.(Met430Val) and green denotes NM_017551.3:p.(Val529Ile). **(B)** Four variants were identified in GluN3A with the red denotes NM_133445.3:p.(Pro108Ala), yellow denotes NM_133445.3:p.(Arg480Gly), green denotes NM_133445.3:p.(Lys488Glu), and blue denotes NM_133445.3:p.(Asp834Asn). NM_133445.3:p.(Pro19Ser) is in the N-terminal signal peptide that is not presented in the mature protein. The 3D structure was generated by the Swiss-Pdb Viewer (Guex and Peitsch 1997).

Two variants in *GRID1* and five in *GRIN3A* were observed in our cohort. The location of the amino acid changes in their corresponding proteins is demonstrated in Figure 4. The distribution of these variants in our cohort and functional predictions by PolyPhen-2 (Polymorphism Phenotyping v2) (Adzhubei et al., 2010), Mutation Taster (Schwarz et al., 2014), and SIFT (Sorting Intolerant From Tolerant) (Sim et al., 2012) as well as variant classification according to the American College of Medical Genetics and Genomics guideline (Richards et al., 2015) are presented in Table 2.

**TABLE 2:** The genetic variants of *GRIND1* and *GRIN3A* with their distribution and functional prediction.

Variants	Patients with ADR (n = 22)	Patients without ADR (n = 61)	Prediction methods			Variant classification according to ACMG guideline
			PolyPhen-2	Mutation taster	SIFT	
*GRIND1*						
*GRID1*:c.1585G>A	5 (22.7)	3 (4.9)	Probably damaging	Disease causing	Deleterious	Benign
*GRID1*:c.1287_1288delinsGG	2 (9.1)	1 (1.6)	N.a	N.a	N.a	VUS
Variants	Patients with behavior ADR (n = 14)	Patients without behavior ADR (n = 69)				
*GRIN3A*						
*GRIN3A*:c.322C>G	1 (7.1)	0 (0.0)	Benign	Harmless polymorphism	Tolerated	VUS
*GRIN3A*:c.55C>T	0 (0.0)	1 (1.4)	Possibly damage	Disease causing	Deleterious	VUS
*GRIN3A*:c.1462A>G	1 (7.1)	3 (4.3)	Benign	Disease causing	Tolerated	Likely benign
*GRIN3A*:c.1438C>G	3 (21.4)	1 (1.4)	Possibly damage	Disease causing	Deleterious	VUS
*GRIN3A*:c.2501_2502delinsCA	2 (14.3)	7 (10.1)	N.a	N.a	N.a	VUS

Categorical variables were presented as n (%).

Abbreviations: ADR, adverse drug reaction; ACMG, american college of medical genetics and genomics; N.a., not available; Polyphen-2, Polymorphism Phenotyping v2; SIFT, sorting intolerant from tolerant; VUS, variants of uncertain significance.

## Discussion

We analyzed the association between the rare variants of the glutamate receptor genes and the outcomes of PER therapy. The gene group burden analysis revealed a statistically significant association between the occurrence of ADR and enriched rare variants of the glutamate gene group. Further analysis revealed the enriched rare variants in the glutamate ionotropic receptor delta subunit had a nominal association with the occurrence of ADR. Furthermore, the gene burden analysis showed that *GRID1* had a nominal association with the occurrence of ADR and *GRIN3A* had a nominal association with the occurrence of behavior ADR. In the end, the nominal association we found did not remain statistically significant after the Bonferroni correction.

Perampanel (PER) is a non-competitive AMPA receptor antagonist (Plosker, 2012), which belongs to one of the glutamate receptors, along with the NMDA receptor, kainite receptor, delta receptor, and metabotropic glutamate receptor (Hansen et al., 2021). These glutamate receptors work in harmony to mediate the excitatory neurotransmission of glutamate (Reiner and Levitz, 2018). The AMPA and NMDA receptors are located in the postsynaptic density at the postsynaptic membrane just opposite the presynaptic vesicle release site and participate in synaptic signal transmission (Scheefhals and MacGillavry, 2018). The metabotropic glutamate receptor was located outside but surrounding the postsynaptic density and served to integrate the function of synapse at high-frequency glutamate release (Rondard and Pin, 2015). The kainite receptor regulates synaptic plasticity with the AMPA and NMDA receptors (Nair et al., 2021). The delta receptors participate in the synaptic organization (Burada et al., 2022) and may alter the number of AMPA receptors in the synapse (Yamasaki et al., 2011). In line with our finding that enriched genetic variants of the glutamate receptor gene group are associated with the presence of ADR. This could be partially explained by the fact that the combined effect of the genetic variants might alter the global interaction of different types of glutamate receptors. These alternations result in changing the overall reaction of the glutamate receptors after PER use and lead to an increase in the risk of ADR in susceptible patients. It is known that PER binds to the AMPA (Yelshanskaya et al. 2016) and kainite receptor (Taniguchi et al. 2022) but our study did not find variants in their corresponding genes, *GRIA1-4* and *GRIK1-5,* respectively, to be associated with PER response through either gene group or gene burden analysis. This could be the result of a relatively small sample size. Genetic variants of glutamate receptors are also linked to epilepsy, such as polymicrogyria is associated with mutations in *GRIN1* (Fry et al. 2018), epilepsy-aphasia spectrum disorders with mutations in *GRIN2A* (Carvill et al. 2013), epilepsy and movement disorders with *GRIN2B* (Platzer et al. 2017), epileptic encephalopathy with *GRIN2D* (Li et al. 2016), and developmental epileptic encephalopathy with *GRIA2* (Salpietro et al. 2019). We did not find the association of PER responsiveness to be related to variants among these genes.

The nominal association of the presence of ADR after PER use with enriched rare variants in glutamate ionotropic receptor delta type subunit as well as *GRID1* suggests the delta receptors could have some role in PER-associated ADRs. Two types of glutamate ionotropic receptor delta-type subunits are identified, glutamate receptor delta 1 (GluD1) and glutamate receptor delta 2 (GluD2) (Burada et al., 2022). Their role is mainly to mediate the synaptic formation and organization, regulate the function of other glutamate ionotropic receptors, and control the trafficking of other glutamate ionotropic receptors (Burada et al., 2022). In the hippocampus, GluD1 enhances the AMPA and NMDA current as knockdown of GluD1 results in the reduction of excitatory synaptic transmission (Tao et al., 2018); in the cerebellar, GluD2 helps the formation of synapses between parallel fibers-Purkinje cells (Yuzaki, 2013). Most interestingly, there was evidence that knock-out of GluD1 decreased the expression of AMPA and NMDA receptors in mice (Yadav et al., 2012; Gupta et al., 2015) and coupling of GluD2 with synaptic organizer complex may trigger the internalization of the AMPA receptor (Elegheert et al., 2016). Therefore, functional alternation from genetic variants of the delta receptor might cause different expression or activity of AMPA and NMDA receptors on the neuronal membrane. This may result in a different response of AMPA receptors when interacting with PER and possibly increase the risk of the presence of ADRs. There is also evidence that GluD is a functional ion channel that interacts with the G protein-coupled glutamate metabotropic receptor (Kumar et al. 2023). They exert a tonic current (Lemoine et al. 2020; Copeland et al. 2023) to maintain the resting membrane potential and activate glutamate metabotropic receptor to promote bursts of action potential (Prisco et al. 2002; Zheng and Johnson 2002). GluD1 is expressed strongly in the cortex, basal ganglia, hippocampus, thalamic, dorsal raphe, and hypothalamic nuclei, while GluD2 in the cerebellum and hypothalamic nuclei (Nakamoto et al. 2020). This wide distribution of GluD1 could explain the association between variants in the *GRID1* and the overall occurrence of ADR. In our cohort, *GRID1*:c.1585G>A was predicted to have functional consequence. There are evidences linking the variants of *GRID1* with schizophrenia and bipolar disorder (Treutlein et al., 2009; Greenwood et al., 2011). *In vivo* study have demonstrated the coupling of glutamate metabotropic receptor 1/5 and GluD1 is essential to the firing of dopamine neurons in the midbrain (Benamer et al., 2018) that are correlated with the physiopathology of schizophrenia (Paladini and Roeper, 2014; Stopper and Floresco, 2015). Although variants of *GRID1* are not linked to behavior ADRs in our cohort, our finding may warrant a further investigation of this variant to elucidate its role in the function of glutamate delta receptors.

Our cohort revealed that *GRIN3A* had a nominal association with behavior ADR. *GRIN3A* encoded the GluN3A subunit of the NMDA receptor (Eriksson et al., 2002). The GluN3A-containing NMDA receptor had a reduction of the NMDA-induced current (Sasaki et al., 2002). This phenomenon usually occurs at the early stage of development, mostly *in utero*, when the GluN3A-containing NMDA receptor serves as a negative modulator to limit or eliminate the formation of weakly connected synapses and stabilized the highly specific and durable synaptic connections (Roberts et al., 2009; Kehoe et al., 2014). The effect of GluN3A on synaptic formation implicated its role in memory and cognitive functions. Genetic studies had linked this subunit to other psychiatric diseases, such as schizophrenia (Takata et al., 2013), nicotine dependence (Yang et al., 2015), and delirium (Kazmierski et al., 2014). In our cohort, one of the *GRIN3A* variants, c.1438C>G, was also found to be associated with schizophrenia (Takata et al., 2013). The roles that GluN3A involved with cognitive function and various psychiatric diseases might make patients harboring variants in *GRIN3A* more susceptible to behavior ADR. Of note, variants among the *GRIN3A* are considered to be more relevant to autism spectrum disorders or schizophrenia (Tarabeux et al. 2011), while *GRIN1, GRIN2A, GRIN2B, and GRIN2D* are more epilepsy-related (Xu and Luo 2018). This may explain why variants of the *GRIN3A* are associated with behavior ADR but not the overall occurrence of ADR.

To our best knowledge, our study is the first to evaluate the association between the rare variants of the glutamate receptor genes and the outcomes of PER treatment. The development of ADRs after PER might occur in 66.5% of the patients but were usually tolerable (Tsai et al., 2018). Most ADRs are associated with high dose or fast titration while dizziness, falls, and behavior ADRs may be prevented by slow titration (Tsai et al., 2018). Bedtime administration also reduces the occurrence of somnolence and dizziness (Takata et al., 2013). Around 5%–15% of patients taking PER may develop irritation, violent behavior, and/or psychotic symptoms (Gil-Nagel et al., 2018; Tsai et al., 2018). These behavior ADRs could be catastrophic to patient care and decrease their quality of life. Another ASM that might cause behavior ADR was levetiracetam (Chen et al., 2017). A pharmacogenomic study of psychiatric ADR and levetiracetam revealed no association in either common or rare genetic variants (Campbell et al., 2022). However, Campbel et al. utilized a hypothesis-free analysis that included a greater number of genes, and no statistically significant results were found after correction for multiple comparisons. Thus, we proposed a hypothesis based on the pharmacokinetics property of PER that variants in the glutamate receptor genes might alter the response of PER. This approach enabled us to find some associations relevant to the pharmacokinetic property of PER. PER mainly acts on AMPA receptors to exert its antiseizure effect (Ceolin et al., 2012). It is currently unknown whether GluD1 and GluN3A interact with the AMPA receptor (Hansen et al., 2021). Perhaps, a future study allocating function MRI to study patients with and without ADR after PER use may reveal the change in brain function, such as that used in the neuropsychiatric studies in patients with systemic lupus erythematosus (Mikdashi 2016). Therefore, the impact of the *GRID1* and *GRIN3A* variants is still largely unknown based on the available literature. Nevertheless, our findings give a glimpse into the possible underlying genetic variants that may affect the response of the drugs.

Although the variants observed in *GRID1* and *GRIN3A* only had a nominal association with the occurrence of ADR and behavior ADR, respectively, the fact that some of the variants are located in NTD is interesting. NTD is the site that regulates the assembly, trafficking, and function of glutamate receptors (Hansen et al., 2021). Currently, there is no functional evaluation regarding the consequences of the variants we found and the exact interactions of different glutamate receptors remain unknown. However, NTD has the important function of forming a trans-synaptic complex to maintain the integrity of synapses by recruiting neurotransmitter receptors and postsynaptic scaffolding proteins (Sheng and Hoogenraad 2007). It is essential in the formation of excitatory and inhibitory synapses (Fossati et al. 2019) and changes resulting from genetic variants of this region could affect the interaction of various receptors. Since PER acts mainly on AMPA receptors, indirect evidence suggests that non-AMPA glutaminergic receptors, such as glutamate ionotropic receptor delta type subunit and NMDA can affect the function of AMPA receptors. Glutamate ionotropic receptor subunit GluD1 affects the expression of AMPA and NMDA receptors in mice (Yadav et al., 2012; Gupta et al., 2015). The AMPA and NMDA receptors aggregate together in the postsynaptic area to maintain synaptic transmission (Scheefhals and MacGillavry, 2018). As a result, functional changes of non-AMPA glutaminergic receptors may alter the response of AMPA receptors to PER and lead to different outcomes.

In our cohort, 16.9% of patients achieved seizure-free for 1 year after the use of PER. A real-world observational study revealed that 7.2% of patients achieved seizure-free after receiving PER for 1 year (Villanuevaet al. 2016b). With a median of four ASMs before PER use in our cohort and a mean of 7.8 ASMs in the work of Villanueva et al., these patients were having drug resistance epilepsy defined by the International League Against Epilepsy (Kwan et al., 2010). This may explain the reason for the low responder rate in both studies. Various mechanisms contribute to drug resistance epilepsy, including the change of structure or expression of the target of ASMs, alteration of the drug delivery system across the brain, the pharmacodynamic modification that reduces the absorption of ASMs, or formation of the abnormal neural circuits (Loscher et al., 2020). Our study only focused on the association of outcome and the genetic variants in the glutamate receptors. Other genetic variants in the drug-transporting system of the CNS or drug metabolism may also contribute to the responsiveness of PER, which warrants further studies. Besides the change caused by genetic variants, the prolonged seizure may change the expression of the ASM target, such that the expression of sodium channels in human hippocampal tissue is decreased in patients with temporal lobe epilepsy (Remy and Beck, 2006). Currently, no evidence has suggested a similar mechanism existed for AMPA receptors (Lukawski and Czuczwar, 2021) to explain the resistance of PER and may be an interesting topic of future research.

Our study had several limitations. First, our cohort only included Taiwanese patients. Our findings might not be able to apply to patients with different ethnic backgrounds. Just as the association of HLA-B*1502 and Stevens-Johnson syndrome was mainly found in Southeast Asian populations (Chung et al., 2004). Second, we had a relatively low number of patients in our cohort. This limited our ability to find an association between the responsiveness of PER and 1-year retention of PER use. A larger cohort will be needed to validate our findings. Despite the small sample group we had, our research did suggest genetic variants in the glutamate receptor gene group were associated with ADR after PER use. Third, our study only examines the impact rare variants have on the responsiveness of PER, common variants may also have contributions. One study regarding the common variants of the AMPA receptors and the response to antidepressants revealed no association between the two (Chiesa et al. 2012). However, the association between the common variants of the AMPA receptor and the responsiveness of PER has not been studied, which may also provide valuable data for the application of predicting the outcome of PER use. In addition, analysis of the common variants requires a larger patient group to achieve adequate statistical power to find any significant difference. Fourth, we only focused on the glutamate receptors, while auxiliary proteins that interact with glutamate receptors could also affect its response during PER use. Transmembrane AMPA receptor regulatory proteins (TARPs), for example, enhance the conductance level of AMPA receptors (Zhang et al. 2014) and alter their pharmacological properties (Tomita et al. 2006). Variants of the TARPs could result in changing the interplay of TARPs and AMPA receptors leading to different responses to PER use. With ongoing study and the advances in genetic analysis methods, we hope the combination of more genetic association data can reach the ultimate goal of providing patients with the most suitable ASM based on genetic testing.

## Data Availability

The datasets generated and analyzed in the current study are available from the corresponding author upon reasonable request.
