# Supplementary material for: A pharmacogenetic study of perampanel: association between rare variants of glutamate receptor genes and outcomes
Source: Front Genet. 2023 Nov 24;14:1215493. doi: 10.3389/fgene.2023.1215493 (PMC10704178; doi:10.3389/fgene.2023.1215493)
Supplement: Supplementary file 1 [file Table1.DOCX]

Supplemental Table 1. The association of seizure types and the outcomes of perampanel use.

| Seizure types | Not respond to PER | Respond to PER | p-value |
| --- | --- | --- | --- |
| Focal | 63 (75.9) | 13 (15.7) | 0.740 |
| Generalized | 3 (3.6) | 1 (1.2) |  |
| Unknown | 3 (3.6) | 0 (0.0) |  |
|  | No ADRs | With ADRs |  |
| Focal | 55 (66.3) | 21 (25.3) | 0.811 |
| Generalized | 3 (3.6) | 1 (1.2) |  |
| Unknown | 3 (3.6) | 0 (0.0) |  |
|  | No behavior ADRs | With behavior ADRs |  |
| Focal | 63 (75.9) | 13 (15.7) | 0.740 |
| Generalized | 3 (3.6) | 1 (1.2) |  |
| Unknown | 3 (3.6) | 0 (0.0) |  |
|  | Not reaching one-year retention | Reached one-year retention |  |
| Focal | 18 (21.7) | 58 (69.9) | 0.104 |
| Generalized | 2 (2.4) | 2 (2.4) |  |
| Unknown | 2 (2.4) | 1 2.1) |  |

Categorical variables were presented as n (%).

Abbreviations: ADR= adverse drug reaction, PER= perampanel

Supplemental Table 2. The association of etiologies of seizure and the outcomes of perampanel use.

| Etiologies | Not respond to PER | Respond to PER | p-value |
| --- | --- | --- | --- |
| Genetic | 7 (8.4) | 0 (0.0) | 0.729 |
| Immunological | 4 (4.8) | 0 (0.0) |  |
| Infectious | 3 (3.6) | 1 (1.2) |  |
| Metabolic | 1 (1.2) | 0 (0.0) |  |
| Structural | 26 (31.3) | 7 (8.4) |  |
| Unknown | 28 (33.7) | 6 (7.2) |  |
|  | No ADRs | With ADRs |  |
| Genetic | 7 (8.4) | 0 (0.0) | 0.141 |
| Immunological | 3 (3.6) | 1 (1.2) |  |
| Infectious | 1 (1.2) | 3 (3.60 |  |
| Metabolic | 1 (1.2) | 0 (0.0) |  |
| Structural | 23 (27.7) | 10 (12.0) |  |
| Unknown | 26 (31.3) | 8 (9.6) |  |
|  | No behavior ADRs | With behavior ADRs |  |
| Genetic | 7 (8.4) | 0 (0.0) | 0.0509 |
| Immunological | 3 (3.6) | 1 (1.2) |  |
| Infectious | 1 (1.2) | 3 (3.6) |  |
| Metabolic | 1 (1.2) | 0 (0.0) |  |
| Structural | 27 (32.5) | 6 (7.2) |  |
| Unknown | 30 (36.1) | 4 (4.8) |  |
|  | Not reaching one-year retention | Reached one-year retention |  |
| Genetic | 4 (4.8) | 3 (3.6) | 0.138 |
| Immunological | 2 (2.4) | 2 (2.4) |  |
| Infectious | 0 (0.0) | 4 (4.8) |  |
| Metabolic | 0 (0.0) | 1 (1.2) |  |
| Structural | 10 (12.0) | 23 (27.7) |  |
| Unknown | 6 (7.2) | 28 (33.7) |  |

Categorical variables were presented as n (%).

Abbreviations: ADR= adverse drug reaction, PER= perampanel

Supplemental Table 3. The association of the interval from seizure onset to the use of perampanel and outcome of perampanel use.

|  | Not respond to PER | Respond to PER | p-value |
| --- | --- | --- | --- |
| The interval from seizure onset to the use of perampanel (years) | 22.0 (9.0-32.0) | 22.0 (3.24-43.75) | 0.836 |
|  | No ADRs | With ADRs |  |
| The interval from seizure onset to the use of perampanel (years) | 21.0 (8.0-30.0) | 35.5 (10.25-39.7) | 0.160 |
|  | No behavior ADRs | With behavior ADRs |  |
| The interval from seizure onset to the use of perampanel (years) | 21.0 (9.0-30.0) | 31.5 (7.8-55.5) | 0.333 |
|  | Not reaching one-year retention | Reached one-year retention |  |
| The interval from seizure onset to the use of perampanel (years) | 21.9 (8.3-31.5) | 22.0 (9.0-34.9) | 0.642 |

Continuous variables were presented as median (interquartile range)

Abbreviations: ADR= adverse drug reaction, PER= perampanel
